# A transformer-based method for the cap analysis of gene expression and gene expression tag associated capping region prediction in RNA

**DOI:** 10.1080/15476286.2026.2629530

**Published:** 2026-02-10

**Authors:** Dibya Kanti Haldar, Avik Pramanick, Chandrama Mukherjee, Pralay Mitra

**Affiliations:** a Centre for Computational and Data Sciences, Indian Institute of Technology Kharagpur, Kharagpur, India; b Department of Computer Science and Engineering, Indian Institute of Technology Kharagpur, Kharagpur, India; c RNABio Lab, Institute of Health Sciences, Presidency University, Kolkata, India

**Keywords:** Cap analysis of gene expression, capping, transcription start site, transformer, Bi-LSTM

## Abstract

5’ RNA capping is one of the major post-transcriptional modifications for the mobility and stability of RNA molecules. Measuring 5’ caps of RNAs can help quantify expression levels of mRNAs and lncRNAs. One of the most successful RNAseq methods that has used capping as a tool to quantify expression of transcription is Cap Analysis of Gene Expression (CAGE). Computational prediction of capping can therefore be used as a precursor to the prediction of transcriptional expression. Unfortunately, there is hardly any computational technique that has focused purely on predicting 5’ capping. We have developed a transformer-based method for computational prediction of capping from DNA sequences. Our Llama and ReLoRA-based pre-training model, and Llama and LoRA-based fine-tuning model predict capping associated regions. We have used Leave-one-chromosome-out-cross-validation for our model. The average accuracy, and F1-score after fine-tuning the human genome hg19 (mouse genome mm9) for sequence classification is 79.12% (78.09%) and 78.11% (76.17%), respectively. We noted attention peak-based motifs having an aggregate Wilcoxon rank-sum p-value of 1.075e-10 between the attention peak region and the entire context window for the predicted positive motifs; an aggregate p-value of 7.17e-18 for the predicted negative motifs; and an aggregate p-value of 6.70e-08 between the attention peaks of the predicted positive and the predicted negative motifs. Our Llama-based approach aims to create a sequence-based framework to identify capping associated regions corresponding to CAGE peaks. Our analysis reveals statistically significant motifs from the regions of peak attention scores, which demonstrates biological relevance for some through their resident sites matching with known TF motifs.

## Introduction

The eukaryotic cell, biological unit of all multi-cellular life, has three components encapsulating all the information and carrying out all major activities, DNA, RNA and proteins. In the process, the pre-mRNAs undergo post-transcriptional modifications to become RNA, and synthesize proteins. The post-transcriptional modifications are polyadenylation, capping, methylation and phosphorylation.

Capping [1,2] is the process of the addition of a 7 methyl-guanosine cap to the 5’ end of all RNA polymerase transcribed RNAs, after which the capped RNA can cross the nuclear membrane and traverse through the cytoplasm for translation. Being a post-transcriptional modification, RNA 5’ capping is essential for the translation initiation, and the prevention of the exonuclease-based degradation of the RNA 5’ region [3]. Decapping of RNA is believed to be destined for degradation. However, this concept is challenged after the identification of cytoplasmic recapping of a subset of mRNAs as well as lncRNAs [4,5]. Recapping can occur at the sites other than Transcription start site (TSS), upstream of the promoter, as evidenced by the capping of decaying RNA and the capping of spliced exons [6,7]. Therefore, the cap trapping protocol would also identify caps at sites downstream of TSS. RNA capping is a regulatable activity, and the status of RNA capping is not fixed. It can fluctuate during different cellular conditions including but not limited to differentiation and development. Well-known methods to identify cap structures include CapIP, CapTure, CapQ, Oligocapping, CapSMART, CapTrapper, CapQuant, and CAGE [5]. New cap structures, i.e. those not based on the 7 methyl-guanosine cap, were identified in human and mouse cells using CapQuant8, and the identified caps are based on alternate enzymes and mechanisms like FAD, NAD+, NADH, UDP-Glc, UDP-GlcNAc, mGpppmA [8–10]. CAGE or cap analysis of gene expression [11,12] is a popular method of RNA sequencing, originally performed by counting the RNA 5’ caps, captured using the cap trapping protocol [13]. CAGE is used primarily for TSS annotation, along with methods like RAMPAGE, GRO-cap, STRT, NanoCAGE, etc. [14,15]. Even so, CAGE analysis has identified that around 25% of the capping sites in mammalian genome were located at non-promoter regions, namely at spliced exons [16].

Conversely, CAGE has only a recall rate of 75% in TSS peak identification [14,15]. Therefore, capping occurs near promoter sites as well as at some sites across the gene, making capping prediction a problem disparate from TSS prediction. The first CAGE protocol involved cap trapping at the 5’ ends, synthesis of cDNA fragments using oligo-dT primers, tag cleaving using restriction enzymes, followed by Sanger sequencing [17]. DeepCAGE [18] added barcode multiplexing to the protocol. Subsequent methods like CAGEscan [19,20] and HeliScopeCAGE [21,22] skipped the enzymatic tag cleavage part of the original protocol. The CAGE protocol generates CAGE tags for each of the sequence fragments it analyzes, and the CAGE peaks are derived from the overlapping tags. The CAGE protocol can identify TSS with a very high precision, and sometimes the CAGE peaks can be down to a single nucleotide, suggesting a single nucleotide precision. CAGE peaks usually range from 1 to 50 bps, and are usually found in the first few hundred kilobases from TSS signals. CAGE peaks frequently overlap with H3K4me3 peaks and DNaseseq peaks [14,15].

The TSS can be signalled by motifs like the TATA box, the Initiator element, the promoter upstream element, and the promoter downstream element. The presence of TSS signals does not by themselves indicate the start of transcription, but a possibility of one. Sometimes TSS signals are present in isolation, and are referred to as sharp TSS signals. Other times, they might be present in a cluster of overlapping or nearby TSS signals, referred to as a broad TSS signal. The TATA box is one of the most prominent TSS signals, and is represented by the pattern TATAWADR, i.e. TATA[A/T]A[G/A/T][G/A]. It is recognized by the Tata Binding Protein (TBP), a general Transcription Factor, and associated with TBP Associated Factors (TAFs) [23]. The Initiator is also a common TSS motif, and is identified by the pattern YYANWYY, i.e. [T/C][T/C]A[A/T/G/C][A/T][T/C][T/C]. The Promoter Upstream Element, usually located upstream of the TATA box motif, is recognized by the pattern SSRCGCC ([G/C][G/C][G/A]CGCC), whereas the Promoter Downstream Element, usually located downstream of the Initiator, is identified by the pattern RGWYV, i.e. [G/A]G[A/T] [T/C][G/C/A].

Differential gene expression is the mechanism responsible for pluripotency in the Eukaryotic organisms. Gene expression is dependent on factors such as the presence of cell type-specific TFs, chromatin accessibility of the gene, methylation state of the underlying histones and of the gene itself, the active enhancers and silencers regulating the gene, the transcription activation and repression mechanisms, regulatory non-coding RNAs. This makes the quantification of gene expression a multifactor problem, many of which can be obtained from the DNA sequence itself, like enhancer and silencer sites. Other factors, like associated TFs, regulatory RNAs, and methylation state of the underlying histones, can be identified using other mechanisms like protein expression for TFs expressed in cell/tissue, gene regulatory networks for regulatory RNAs and ChIP seq for analysing histone modifications. While this makes it difficult to identify the active TSS from sequence alone, there have been attempts to identify them from sequence alone.

Capping sites, on the other hand, can be identified from DNA sequence alone, even though attempts to predict capping sites have been scarce. While the CAGE protocol maps the capped RNA, the CAGE peaks are often clustered and not found in the same sites consistently, with the average deviation between nearby CAGE peaks being 51 nucleotides; reducing the reliability of the CAGE peaks as narrow capping sites. Increasing the context length to incorporate more DNA around CAGE peaks would allow us to reliably predict broad capping sites. However, CAGE peak sites are not found to be associated with any known motif, and TSS motifs like promoters and initiators are usually present at a variable distance from the CAGE peaks. In large context of 500 or 1000 bps, sequences in possession of CAGE peaks as well as sequences without have been found to possess similar number of TSS signals in them.

We have developed a framework that takes the DNA subsequence as input and outputs the predicted capping sites. Our framework first divides the input DNA sequence into words of size 8 nucleotides where each word shares the first 4 nucleotides with the previous and the next 4 nucleotides with the next. We repeated this process with offsets of 1, 2 and 3, before passing it through a Byte Pair Encoding-based Tokenizer, and to prevent motifs from being missed out. The tokens are then passed through our Llama [24] and ReLoRA [25] based pre-training module using a learning rate scheduler. The pre-training is followed by a Llama and LoRa [26] based fine-tuning. We have trained our model using Leave-one-chromosome-out-cross-validation (LOCOCV) [27], using a 1:1 proportion of positive and negative samples, in order to prevent data leakage and conform to standard dataset creation practices. Our model achieved an LOCOCV capping classification accuracy of 79.12 ± 1.16% (78.25 ± 1.00%), a precision of 78.64 ± 2.10% (79.84 ± 3.50%), and a recall of 77.66 ± 2.11% (73.49 ± 3.275%), along with an F1 score of 78.11 ± 1.13% (76.40 ± 1.01%) on the human (mouse) genome using only Llama and LoRa-based fine-tuning on our pre-trained model for a context length of 512.

## Materials and methods

Problem Definition: The work intends to predict CAGE Tag associated regions, indicative of capping, using machine learning-based methods, and corroborate the regions with nearby TF binding sites. Input is a nucleotide sequence consisting of [ATCG nucleotides] of size 512 or 1024 bases. Our method takes a nucleotide sequence and returns the presence or absence of capping associated regions in that sequence.

To obtain the positively labelled sequences, we made sure that the CAGE Tag was part of the sequence. The positively labelled sequences were the sequences derived from CAGE Tag start position ± CONTEXT_WINDOW × 0.5, where the CONTEXT_WINDOW was a fixed size, typically 512 or 1024.

The negatively labelled sequence is randomly situated between 10k nucleotides and 20k nucleotides away from a CAGE Tag. The negatively labelled sequences were the sequences derived from sequence start position + CONTEXT_WINDOW.

The prediction function is:

(1) $$f\left(x\right)\;=\;\mathrm{LoRA}\left(\mathrm{LlamaForSequenceClassification}\left(\mathrm{ReLoRA}\left(\mathrm{LlamaForCausalLM}\left(x\right)\right),x\right)\right)$$

## Dataset details

We created deep learning techniques for 5’ cap detection by training the models on the human genome hg19 and mouse genome mm9. The FANTOM5 [28] consortium has bed files containing CAGE peaks for the human genome hg19 and the mouse genome mm9. The CAGE peaks represented the genomic locations of the capping sites found through CAGE. We downloaded the human genome hg19 and the mouse genome mm9. For creating the dataset, we downloaded the hg19 and mm9 fasta sequences adjacent to the CAGE peaks, downloaded from FANTOM5, which included a near equal number of nucleotides upstream and downstream to the peak. For our site prediction task, we divide the genomic sequence into fixed size subsequences and then apply binary classification on the subsequences. Positive sequences were those which contained CAGE peaks, whereas negative sequences had the CAGE peaks at a considerable distance away from them [29]. The number of positive and negative sequences obtained from the human genome hg19 and the mouse genome mm9 are given in Table 1.

**Table 1.** Chromosomes and corresponding number of positive (pos) and negative (neg) samples (hg19 and mm9). The hg19 positive sequences return the number of samples per chromosome in our dataset having CAGE peaks taken from the hg19 human genome. The mm9 positive sequences return the same from the mm9 mouse genome. The hg19 negative sequences and mm9 negative sequences return the number of samples per chromosome in the dataset not having CAGE peaks taken from hg19 and mm9 genomes respectively.

Chromosome	Human (hg19)		Mouse (mm9)		Chromosome	Human (hg19)		Mouse (mm9)	
	Pos	Neg	Pos	Neg		Pos	Neg	Pos	Neg
**1**	18654	20923	10089	11284	**13**	4145	4783	6005	6750
**2**	16437	18028	12369	13580	**14**	7031	7586	6201	6769
**3**	11514	12845	8069	8858	**15**	6417	7245	6439	7046
**4**	8963	10035	8714	9721	**16**	6832	7390	5531	6115
**5**	9953	11169	9645	10918	**17**	10370	11323	7537	8241
**6**	11479	12518	9488	10571	**18**	3648	4295	4278	4948
**7**	10410	11700	11803	13057	**19**	10315	11124	5938	6523
**8**	8608	9698	7536	8654	**20**	5483	6275	0	0
**9**	7669	8396	8641	9330	**21**	2563	2778	0	0
**10**	8747	9672	7789	8631	**22**	3873	4023	0	0
**11**	11149	12282	12475	13744	**X**	5789	6453	4303	4819
**12**	10993	12418	5721	6557	**Y**	207	218	32	29

We constructed the positive samples by taking the DNA segments containing the CAGE peaks, as well as 256 nucleotides upstream and downstream of the CAGE peak start sites. We built the negative samples by taking 512 nucleotides long DNA segments randomly between 10,000 bps and 20,000 bps upstream of the CAGE peak start sites, while taking care that these segments do not overlap with the DNA segments in the positive samples. To avoid any misinterpretation, we had to remove those positive samples whose DNA segments had overlapping base pairs with some negative sample, resulting in approximately 10% greater number of negative samples per chromosome than the corresponding positive ones.

## Methodology

We incorporated llama, LoRA and ReLoRA. Llama is a transformer-based model capable of making predictions on long context textual data. It incorporates rotary positional embeddings which encode both the absolute and relative position of the tokens by rotating their corresponding vectors by angles proportional to the position of the tokens in the sequence. Llama also uses grouped multi-query attention where the heads are grouped into sets with each set having different queries but the same value and key for all heads in the set, balancing the efficiency of multi-query attention with the accuracy of multi-head attention. Recent article [30] detects HCC from cfDNA end-motifs using instruction tuned LLMs like llama.

LoRA is shorthand for Low Rank Adaptation, and as can be inferred from the name, its weight update matrix is decomposed into the low rank matrices A and B. If the pre-trained matrix has dimensionality *d*×*k*, then the dimension of matrix A is *d*×*r* and that of the matrix B is *r*×*k*, where the matrix product AB is the proxy for the weight updation during the fine-tuning step, and r has a rank much smaller than either d or k. During LoRA fine-tuning, the matrices A and B are updated, and after updation the proxy weight update is computed by multiplying matrices A and B. LoRA is useful as due to its replacement of the weight updation matrix with low ranked matrices, the matrix multiplication complexity is reduced and since our approach uses llama models with linear layers part of the query, key and value matrix computation, we prefer to use LoRA during fine-tuning and as part of ReLoRA pre-training in order to reduce the resource utilization as much as possible.

ReLoRA is a pre-training algorithm that uses LoRA with restarts. It is initialized with a warm start where for some initial steps, the training is carried out without using LoRA or low rank decomposition of the update matrices. ReLoRA is only applicable to the linear layers in the neural network. After a fixed number of steps, ReLoRA updation is applied to the linear layers which include a LoRA updating, aiming initialization, pruning, and a fixed number of warmup steps.

**Figure 1.**
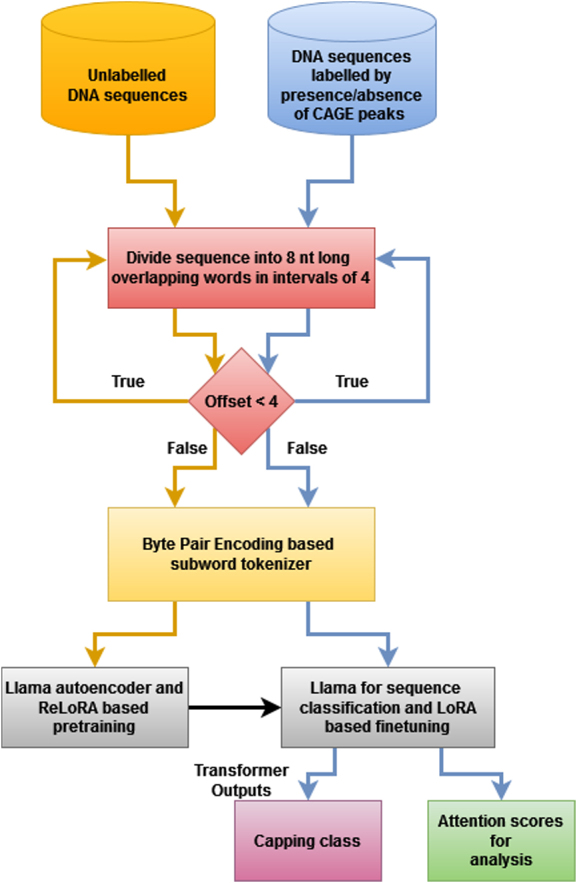
Flow diagram of our approach, showing DNA sequences divided into words, tokenized using Byte Pair Encoding based tokenizer, pre-trained (orange arrows) using Llama and ReLoRA-based pre-training, and fine-tuned (blue arrows) using Llama and LoRA-based fine-tuning. Please note the processes remain same, pre-training and fine-tuning varies in input and output.

Our pre-training dataset was created by combining the genomes of Homo Sapiens (human), Mus Musculus (mouse), Pan Troglodytes (chimpanzee), and Pan Paniscus (bonobo), and dividing them into fragments of size 512 for a context window of size 512. Our approach, as shown in the flow diagram in Figure 1, used Eleuther AI’s GPTNeoX-20B tokenizer, a tokenizer trained on the diverse and extensive PILE dataset [31], which is based on Byte Pair Encoding based sub-word tokenization. Even though our method takes the trained GPTNeox Tokenizer, our approach has an explicit tokenization step which regenerates the sub-word tokens from the nucleotide words generated in a pre-tokenization step using the BPE tokenization strategy. Byte Pair Encoding has characteristics similar to the merge sort algorithm, and starts the tokenization process from tokens of size 1, in other words, character level tokens. The most frequent token pair from all pairs of adjacent tokens is taken and concatenated into a single token. This is repeated for *k* number of steps, and ends up building the vocabulary. This tokenization is able to obtain most of the prefixes, stem words and postfixes as tokens when trained on the English text corpus.

We adapted the BPE tokenization process [32] as follows:

(2) $$V={\left(w_{j},f_{j}\right)}_{j=1}^{N}w_{j}=\left(s_{j,1},s_{j,2},\ldots ,s_{j,L_{j}}\right)$$

where, ***w*** refers to the word token, ***f*** refers to its frequency, ***N*** is the number of unique tokens, ***S*** is the sequence, ***w***_***j***_ refers to the *j*^th^ word, and ***L***_***j***_ refers to the length of the *j*th’ word.

We compute the frequency (***C***) of the tokens *a* and *b* found together in the corpus

(3) $$C\left(a,b\right)=\underset{w,f\in V}{\sum }f\overset{\left|\left(w\right)\right|-1}{\underset{i=1}{\sum }}1\mathrm{if}\left(w_{i}=a\right)\wedge \left(w_{i+1}=b\right)$$

Obviously, the largest value of corpus frequency can be computed using

(4) $$\left(a^{*},b^{*}\right)=\mathrm{argmax}\left(a,b\right)C\left(a,b\right)$$

(5) $$\mathrm{Merg}e_{\left(a,b\right)}=\mathrm{ab}V_{t+1}=\left(\mathrm{Merg}e_{a^{*},b^{*}}\left(w\right),f\right)\forall \left(w,f\right)\in V_{t}$$

Now, we replace each occurrence of (a*,b*) with (a*b*) and recompute *C* for *k* steps.

We divide DNA sequences of size 512 nucleotides into overlapping tokens of size 8. We repeat the tokenization for offsets of 1, 2 and 3, and append them to the list of tokens. The tokens are then encoded with the BPE tokenizer in creating a vocab of size 50,254.

During the pre-tokenization step, the sequence is divided into subsequences of 8 nucleotides, which we refer as words, where every word other than the first and the last, shares the last four nucleotides with the next word, and the first four nucleotides with the previous word. The first word shares the last four nucleotides with the second word, and the last word shares the first four nucleotides with the second-last word.

(6) $$W_{k}=\;\left[S_{4k},\;S_{4k+1},\;\ldots ,\;S_{4k+7}\right]$$

where S is the sequence, k is the word number, and W_k_ is the word.

In the pre-tokenization step, after the overlapping subsequence or word generation, the entire sequence is repeated three more times with different offsets of 1, 2 and 3. Together with overlapping subsequences, this paradigm achieves an effect similar to 8-mer encoding, but doesn’t introduce unnatural tokens, when words are concatenated.

After adding offsets, W_k_ generalizes to W_k,i_ where,

(7) $$W_{k,i}=\;\left[S_{4k+i},\;S_{4k+1+i},\;\ldots ,\;S_{4k+7+i}]\;\mathrm{where}\;i=[0,1,2,3\right]$$

Next, we pre-trained our pre-training dataset on base 28.17 million parameter LlamaForCausalML model for 1000 steps, before switching to ReLoRA optimized 31.39 million parameters LlamaForCausalML model for a further 13,197 steps. The Llama model had 28.17 million parameters and comprised a positional embedding layer of size 50,254 × 256, 4 heads and thus a 4× llama decoder layer, each comprised of 4 projections: query, key, value and output, followed by Llama Rotary Embedding, and a Llama MLP comprised 3 projections: gate projection, down projection, and up projection.

The Llama model used by us is:

(8) $$\mathrm{LlamaAttention}\;\left(Q,K,V\right)\;=\;\mathrm{softmax}\;\left(\left(Q{\;}_{\mathrm{RoPE}}{K^{T}}_{\mathrm{RoPE}}\right)/\surd \;d_{k}\right)\;V$$

Here, Q, K and V refer to the vector projections of the Query, Key and Value weight matrices. ‘d_k_’ is the dimension of the Key matrix.

For a token at position p, the hidden state be x_p_, then Q_p_ = x_p_W_q_, K_p_ = x_p_ W_k_ , and V_p_ = x_p_ W_v_

The Rotary Positional Encodings [33] were computed as:

(9) $$\theta_{i}=10000^{\left(-2i/d\right)}\phi_{p,i}=p\cdot \theta_{i}$$

θ_i_ is the angular representation of the token at index i, which is then scaled using its absolute position p, and then the Query and Key vectors are rotated as much as p.θ_i_.

(10) $$Q_{p,2i}^{\mathrm{RoPE}}=Q_{p,2i}\cos\left(p\theta_{i}\right)-Q_{p,2i+1}\sin\left(p\theta_{i}\right)i=0,1,\ldots ,2d-1$$

(11) $$Q_{p,2i+1}^{\mathrm{RoPE}}=Q_{p,2i}\sin\left(p\theta_{i}\right)+Q_{p,2i+1}\cos\left(p\theta_{i}\right)i=0,1,\ldots ,2d-1$$

(12) $$K_{p,2i}^{\mathrm{RoPE}}=K_{p,2i}\cos\left(p\theta_{i}\right)-K_{p,2i+1}\sin\left(p\theta_{i}\right)i=0,1,\ldots ,2d-1$$

(13) $$K_{p,2i+1}^{\mathrm{RoPE}}=K_{p,2i}\sin\left(p\theta_{i}\right)+K_{p,2i+1}\cos\left(p\theta_{i}\right)i=0,1,\ldots ,2d-1$$

The Llama attention was followed by Llama MLP specified as below:

(14) $$\mathrm{LlamaMLP}\left(x\right)=\mathrm{Wdown}\left(\mathrm{SiLU}\left(\mathrm{xWgate}\right)⊙\left(\mathrm{xWup}\right)\right)$$

LoRA factorizes weight updates into low-rank matrices, allowing the model to use fewer total updates, and thus lower trainable parameters, during fine-tuning. ReLoRA extends this mechanism to the pre-training step, allowing the complete model to be trained using less GPU memory after training the full model for a few warm-up steps. Consequently, ReLoRA linear layer replaced all the linear projection layers in our llama-based model.

We have used ReLoRA training algorithm [25]:

(15) Wi←Wi+sWi,AWi,B

During the LoRA steps, the weight matrix W_i_ is updated using a scaled product of the low-rank learnable matrices W_i,A_ and W_i,B_.

(16) $$\mathrm{Wi},A\leftarrow \mathrm{kaiminginit}\left(\mathrm{Wi},A\right);\mathrm{Wi},B\leftarrow 0$$

During the ReLoRA warm ups, the Weight matrix W_i,A_ is initialized using Kaiming initialization and W_i,B_ is zero initialized.

The number of CPU threads drops during the ReLoRA cycles, peaking during the warmup steps. Supplementary section ReLoRA shows a considerable decline in the number of CPU threads used during the ReLoRA pre-training process.

The training loss and the validation loss drops continuously in lockstep, with the training loss reaching 0.25195, down from its peak at 11.0 at the beginning, and the validation loss reaching 0.22348, down from its peak at 5.36977 at step 200, when it was first recorded. Only the model tokenized using offsets was able to break below the loss of 1.00, whereas the models tokenized without using offsets failed to do so.

The pre-training loss function is described below:

(17) zb,t=logitsb,t,t=1,…,T−1;yb,t=labelsb,t+1,t=1,…,T−1

(18) $$L=-\left(1/N\right)\sum^{i}=1N\log\left(\exp\left(\mathrm{zi},\mathrm{yi}\right)/\sum^{j}=1\mathrm{Vexp}\left(\mathrm{zi},j\right)\right)$$

where T is the number of tokens in the sequence, V is the vocabulary size, and N is the batch size.

We followed this by tokenizing our training and validation datasets, which would be used during fine-tuning. We tokenized our training and validation datasets using the same tokenizer as during pre-training, having the same vocab of 50,254 tokens. Our datasets were fine-tuned using LlamaForSequenceClassifiation, involving 16 million parameters model for 5 epochs and 23,440 steps. The fine-tuning used LoRA and consisted of 4 attention heads, each comprising self-attention with LoRA query, key, value, and output projections, and 4 hidden layers of LoRA MLP down projection, gate projection, and up projection.

The Llama-based fine-tuning model used the following loss function:

(19) $$L=-\left(1/N\right)\sum^{\log}\left(\exp\left(\mathrm{zi},\mathrm{yi}\right)/\left(\exp\left(\mathrm{zi},0\right)+\exp\left(\mathrm{zi},1\right)\right)\right)$$

where y_i_ ∈ (0,1) and N is the batch size.

## Results

### Training and validation

Our Llama and ReLora-based pre-trained and fine-tuned capping prediction model was trained using LOCOCV on the human genome hg19. It was pre-trained on DNA sequences having a context length of 512, using a Llama transformer having 20 million parameters. It was trained on the original Llama model for 1000 steps, and further trained on the ReLoRA-based Llama model for another 13,197 steps. After fine-tuning the human genome hg19 on llama for sequence classification for 5 epochs on a batch size of 64 for 23,440 steps, we obtained an accuracy of 79.12 ± 1.16%, a precision of 78.64 ± 2.10%, a recall of 77.66 ± 2.11%, and an F1 score of 78.11 ± 1.11% on 24 chromosomes of the human genome using LOCOCV (Figure S1). Similarly, after fine-tuning the mouse genome mm9 on llama for sequence classification for 5 epochs on a batch size of 64 for 23,075 steps, we obtained an LOCOCV accuracy of 78.09 ± 1.08%, a precision of 79.78 ± 3.32%, a recall of 73.105 ± 3.36%, and an F1 score of 76.17 ± 1.23% (Figure S2).

A bigger context window allows us to find more motifs in the promoter neighbourhood, such as the Promoter Downstream Element, Promoter Upstream Elements, silencers, and enhancers. A smaller context window means access to less information about the neighbourhood of the capping sites. The capping predictions are validated using the CAGE peaks, which though not the exact capping sites, usually are shifted by some variable number of nucleotides, since the CAGE protocol measures the CAGE tags in bulk, and the capping sites of different cells usually do not agree. The returned CAGE tags are not located near known TSS signals like TATA boxes and Initiators and can be found at various distances from the TSS signals, with the average distance being around 260 for TATA boxes and 91 for initiator signals. The CAGE peaks also keep shifting and are not found in a common location, since we have found multiple CAGE peaks in the same genomic neighbourhood.

We also searched for differentiating capping motifs by comparing the vocab generated by the attention peaks of both predicted classes, and we obtained a few of them. Since the TSS motifs like TATA box, initiator and the Promoter Downstream Element typically contain eight nucleotides, we too compared the vocabs using nucleotide motifs of length 8. We compared the distribution of these motifs between the entire context window and the attention peak window. Using the Mann-Whitney U test, also known as the Wilcoxon rank-sum test, we obtained the *p*-value of the normalized attention peak motif frequencies against the normalized motif frequencies in the full context window for the predicted positive samples as 1.0753e-10. For the predicted negative samples, we got the p-value as 7.1697e-18. We also compared the motif frequencies between the attention peaks of the predicted positive samples and the predicted negative samples, and obtained the p-value as 6.6977e-08.

**Figure 2.**
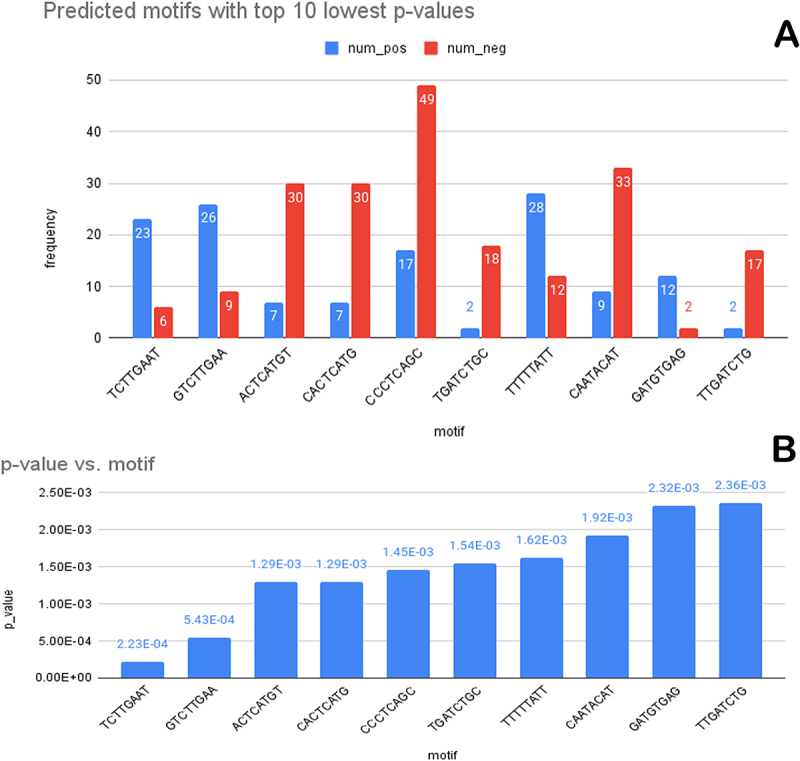
A chart showing (A) predicted motifs with the top 10 lowest p-values for positive class (blue) and negative class (red), and their respective frequencies in regions of peak attention scores, and (B) the corresponding *p*-values for the 10 motifs shown in (A).

After normalizing by the ratio of total vocab frequencies in the positive and the negative classes, we computed the motif-wise p-values of the predicted positive samples by comparing the token count vectors of the predicted positive samples with that of the predicted negative samples. In an order of increasing p-value, the top 10 motifs were TCTTGAAT, GTCTTGAA, ACTCATGT, CACTCATG, CCCTCAGC, TGATCTGC, TTTTTATT, CAATACAT, GATGTGAG, TTGATCTG. These predicted motifs along with their p-values, and class-wise frequencies, found in the regions having the highest attention scores, are shown in Figure 2. There are six more predicted motifs with p-values less than 0.01, namely AATACATA, ATTTAGGG, TAATTTCA, CTTGAATG, ATTCATAG, and ATGTGAGT.

**Figure 3.**
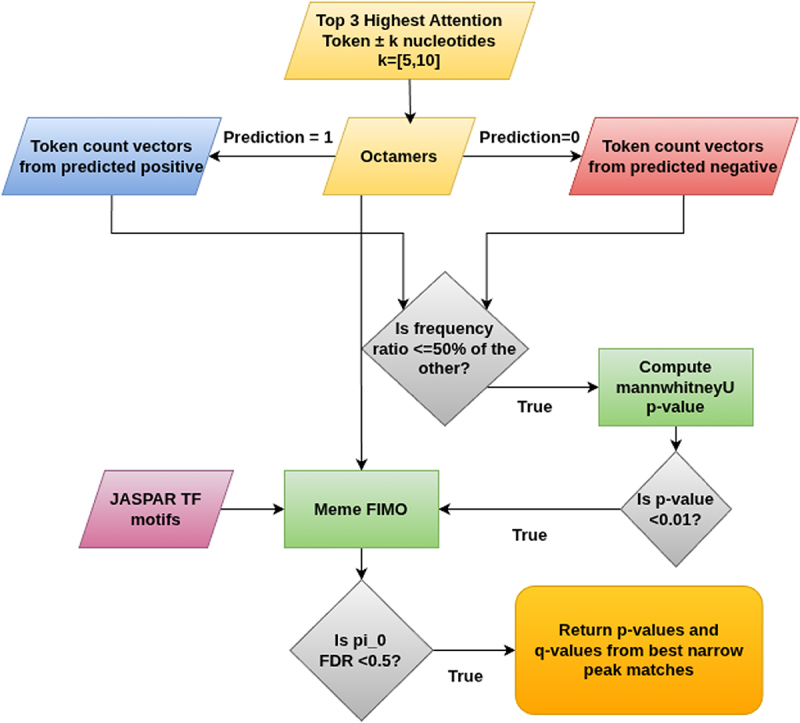
Flow diagram of the process of finding motifs from highest attention tokens. Starting from the top 3 highest attention tokens, we first obtain the highest attention vocab for the positive and negative predicted samples, and from the token count vectors of the octamers in the vocab, we compute the Mann Whiteny U p-values, and filtering for less than 0.01, we match the sequence motifs with the JASPAR TF motifs using FIMO, and return the corresponding *p*-values and q-values for those with pi0 FDR less than 0.5.

One of the above motifs TTTTTATT is found to match with the binding sites of the TF motifs MA0497.1, MA0773.1, MA0846.1, MA0846.1, and MA0052.4 using only the attention token ±5 nts. TTTTTTATT is associated with MA0497.1 with an FDR of 18.43%, a p-value of 3.79e-5 and a q-value of 1.42e-4. It is associated with MA0773.1 with an FDR of 13.95%, a p-value of 6.52e-5 and a q-value of 2.18e-4. It is associated with the TF motif MA0846.1 with an FDR of 24.56%, with 3 matches all having p-values of 2.2e-5 and q-values of 3.03e-4, and 6 matches all having p-values of 7.78e-5 and q-values of 3.57e-4. It is also associated with the TF motif MA0052.4 with an FDR of 15.68%, a p-value of 2.26e-5 and a q-value of 7.24e-5. When looking at the predicted sequence motifs using the attention token ±10 nts, we get ATGTGAGT with a prediction-based p-value of 0.00786. It matches the binding site of the TF motifs MA0601.1, MA0135.1 and MA0791.1. It is associated with MA0601.1 with an FDR of 41.58%, a p-value of 8.95e-6 and a q-value of 4.65e-4. It’s associated with MA0135.1 with an FDR of 43.58%, and a q-value of 5e-4. For 2 matches, the p-values are 7.95e-6 and for the other 12 matches, the p-values are all 1.53e-5. It is also associated with the TF motif MA0791.1 with an FDR of 48.37%. For 10 of the matches, the p-values are all 4.1e-7 and the q-values are all 1.9e-5. For the 2 remaining matches, the p-values are 1.96e-6 and the q-values are 5.09e-5. These associations showcase the statistical significance of the predicted motifs. The process to find these motifs is elaborated in Figure 3.

### Ablation studies

We found that pretraining using a dataset without offsets had a higher pretraining loss than while using a dataset with three offsets 1, 2 and 3. The validation dataset loss during pretraining reached 0.23 after 10,000 steps when the dataset has 3 offsets, compared to a pre-training validation set loss of 0.52 using 1 offset and a pre-training validation set loss of 1.016 using no offset. Though the offsets had little to no impact during fine-tuning. During fine-tuning, the 0 offset method reported an accuracy of 79.05%, a precision of 78.82%, a recall of 76.93%, and an F1-score of 77.87%. The 1 offset model reported an accuracy of 77.95%, a precision of 77.01%, a recall of 76.93%, and F1-score of 76.97%. Moreover, the three offset models obtained an accuracy of 79.26%, a precision of 79.265%, a recall of 76.34%, and an F1-score of 77.77%. These results are summarized in Table 2. We see here that the model with 0 offsets reported the highest F1-score, whereas the model with three offsets reported the highest accuracy, whereas the model with one offset reported lower values of all the metrics, showing no correlation between the number of offsets and the validation set metrics during fine-tuning, even though the validation loss during pre-training showed an inverse correlation between number of offsets and validation loss. This demonstrates the ability of Byte Pair Encoding of finding the relevant tokens, even without providing the alternate words with different starting nucleotides, similar to the concept of *k*-mers.

**Table 2.** Comparison between metrics of llama + ReLoRA models trained on hg19 and validated on left out chromosome 1, using different number of offsets.

Number of offsets	Accuracy	Precision	Recall	F1-score
0	79.05%	78.82%	76.93%	77.87%
1	77.95%	77.01%	76.93%	76.97%
3	79.26%	79.26%	76.34%	77.77%

The llama-based model when trained using a bigger context window of 1024, leaving out chromosome 1 as the validation dataset, obtained an accuracy of 79.735%, a precision of 75.72%, a recall of 83.52%, and an F1 score of 79.425%. Whereas when trained using a smaller context window of 256, similarly leaving out chromosome 1 for validation, attained an accuracy of 75.5%, a precision of 76.74%, a recall of 71.89%, and an F1-score of 74.24%. While using a context window of 512, similarly leaving out chromosome 1 for validation, we obtained an accuracy of 79.26%, a precision of 79.26%, a recall of 76.34%, and an F1-score of 77.77%. This showed us that using a bigger context window consistently increases the performance metrics of our method, as can be seen in Table 3. We obtained the LOCOCV metrics for the human genome hg19 when trained using a context window of 1024 and obtained an average accuracy of 79.52 ± 1.17%, an average precision of 79.03 ± 2.04%, an average recall of 77.72 ± 32%, and an average F1-score of 78.33 ± 1.21%, as shown in Figure S4. This showed us that on an average, using a context length of 1024 provides us a 0.4% advantage in accuracy and a 0.2% advantage in F1-score than using a context length of 512. Trying out even larger context windows would require more GPU resources. The above results are compiled in Table 3.

**Table 3.** Comparison between metrics of llama + ReLoRA models trained on hg19 and validated on left out chromosome 1, using different lengths of context windows.

Context length	Accuracy	Precision	Recall	F1-score
256	75.50%	76.74%	71.89%	74.24%
512	79.26%	79.26%	76.34%	77.77%
1024	79.74%	75.72%	83.52%	79.43%

### Comparisons

We had created a transformer and bi-LSTM-based model for our task. In this model, the positional embeddings were created and passed to the transformer layer, which was then passed to two parallel biLSTM layers, which were then concatenated and passed through another biLSTM layer and a dense layer for prediction. Before creating the positional embeddings, the tokens were encoded using either a 6mer encoding or entropy and FFT-based alignment-free encoding for words of size 20 nucleotides each. Upon training the transformer and bi-LSTM-based model on 24 chromosomes of the human genome hg19, it returned an average Leave-one-chromosome-out-cross-validation (LOCOCV) accuracy of 74.43 ± 1.51%, an average precision of 77.38 ± 5.51%, and an average recall of 67.65 ± 7.97%, along with an average F1 score of 71.62 ± 3.37% for a context window of 512. For a context window of 1000, the model trained on hg19, returned a chromosome 1 accuracy of 72.83%, a precision of 72.58%, a recall of 68.15%, and an F1-score of 70.30%. On the mouse genome mm9, the transformer and bi-LSTM-based model attained a chromosome 1 accuracy of 71.88%, a precision of 83.87%, a recall of 50.04%, and an F1-score of 62.65%, using a context window of 1000 nucleotides.

Also, we have trained a transformer only model for capping prediction. After the creation of positional embeddings, the tokens were passed to a transformer layer, and then to four parallel transformer blocks, each composed of a transformer layer and a global average pooling layer following it. These four were then concatenated two at a time, resulting in two parallel layers. Each of the two parallel layers was then followed by a dense layer, and then concatenated again. Then this layer was followed by a dense layer and the output prediction layer. On our transformer only model, the chromosome 1 accuracy on the hg19 genome using a context window of 1000 nucleotides was found to be 72.24%, the precision was 77.86%, the recall was 57.52%, and the F1-score was at 66.12%. Using a context window of 500, our transformer model obtained a validation accuracy of 70.93%, a precision of 79.64%, a recall of 53.34%, and an F1 score of 63.89%.

The transformer and biLSTM-based model, when trained using a neural network trained embedding layer instead of the alignment free contextual embeddings the convergence stalled and likely got stuck in a vanishing or exploding gradient. However, modifying the input sequence to include 6-mers and completely removing the neural network trained embedding layer yielded an accuracy of 67.20%, a precision of 62.41%, a recall of 94.8%, and an F1-score of 75.27%. It was the first model that we tried, and following a stalled training convergence, we abandoned the neural network trained embeddings in favour of alignment free embeddings.

**Figure 4.**
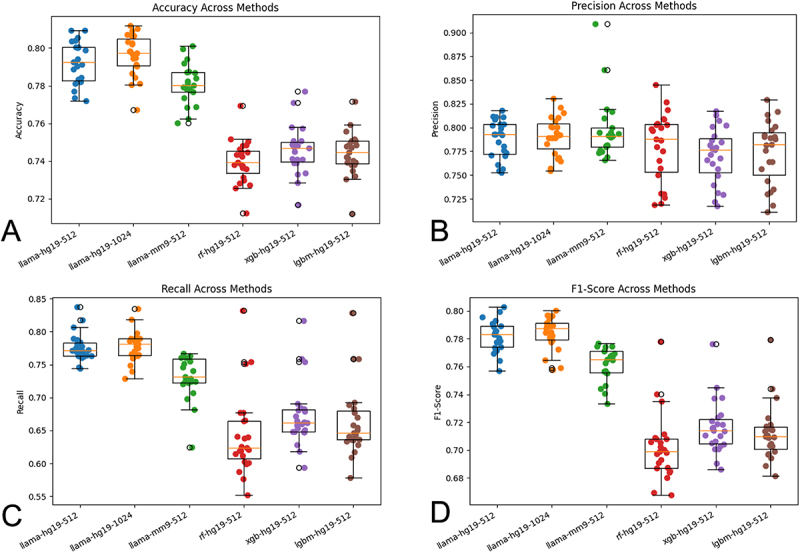
Box plot comparison of (A) accuracy (B) precision (C) recall and (D) F1-score across all the main methods. Along the X-axis, we used the convention: MethodName-genome-contextWindowsize. Method names are: llama & ReLoRA based, random Forest, XGBoost, and LightGBM. Genome is either human (hg19) or mouse (mm9) and the context window is either 512 or 1024.

We also trained an XGBoost-based method from 64 element feature vectors, with the elements representing the frequency of the 3-mers found within the nucleotide sequence. The labels were kept the same as in TBCP. Using LOCOCV, the XGBoost yielded an accuracy of 74.49 ± 1.31%, along with a precision of 76.86 ± 3.05%, recall of 67.47 ± 4.84% and F1-score of 73.68 ± 1.89%. The LightGBM-based model, trained using the same dataset, returned an LOCOCV accuracy of 74.35 ± 1.18%, precision of 77.18 ± 3.46%, recall of 66.69 ± 5.58% and F1 score of 71.30 ± 2.00%. The RF-based model, also trained using the same dataset, returned LOCOCV accuracy at 73.89 ± 1.16%, precision of 77.70 ± 3.85%, recall of 64.70 ± 6.475% and F1 score of 70.27 ± 2.37%. The ML-based or RF, HGB, or LGBM-based methods were first count vectorized using 3grams and then passed through the classifiers. The ML-based models shown above have been compared in Figure S3. And the LOCOCV metrics for the llama and ReLoRa-based model trained on the human genome hg19 using a different context window of 1024 is shown in Figure S4. Figure 4 provides a macro view of the metrics from the llama-based and the machine learning-based models. Table 4 summarizes the comparison metrics among the methods showcased above.

**Table 4.** Leave-one-chromosome-out-cross-validation (LOCOCV) comparison of different deep learning methods used for capping classification in human genome hg19 (except for the last row, which is on the mouse genome mm9). Context length for all the methods is 512, except for the last two rows where it is **1024**. Transformer bi-LSTM uses freq. embeds.

Method	Accuracy(%)	Precision(%)	Recall(%)	F1-score(%)
Transformer bi-LSTM	74.43 ± 1.51	77.38 ± 5.50	67.65 ± 7.97	71.62 ± 3.37
Random Forest	73.89 ± 1.16	77.70 ± 3.85	64.70 ± 6.48	70.27 ± 2.37
LightGBM	74.35 ± 1.18	77.18 ± 3.46	66.69 ± 5.58	71.30 ± 2.00
XGBoost	74.49 ± 1.31	76.86 ± 3.05	64.70 ± 6.48	73.68 ± 1.89
Llama & ReLoRA based	79.12 ± 1.16	78.64 ± 2.10	77.66 ± 2.11	78.11 ± 1.11
**Llama & ReLoRA based**	76.17 ± 1.23	79.03 ± 2.04	77.72 ± 2.32	78.33 ± 1.21
**Llama & ReLoRA based**	78.09 ± 1.08	79.78 ± 3.32	73.11 ± 3.36	76.17 ± 1.23

### Hyperparameter tuning

We trained each of the architectures through five epochs, optimized using the Adam optimizer with beta1 0.9, beta2 0.999, and epsilon 1e-08. We used a batch size of 64 and a linearly reducing learning rate of starting at 5e-5 for training the fine-tuned model. Using a lower batch size of 16 reported lower values in all validation. During validation on left out chromosome 1, a batch size of 64 reported an accuracy of 79.26% against an accuracy of 77.2% for batch size of 16, a precision of 79.26% against 76.645%, a recall of 76.34% against 75.365%, and an F1-score of 77.78% against 76.0% respectively. For pre-training, we had a batch size of 8, and used a learning rate scheduler, which first linearly increases the learning rate, but follows it up by reducing the learning rate on a sinusoidal curve, with downward spikes at fixed intervals.

## Conclusion

CAGE sequencing has been used to measure the transcription expression levels of capped RNAs like mRNAs and lncRNAs. CAGE has been used as a longstanding tool to identify 5’ capping sites, but computational methods to predict capping sites have been scarce so far. CAGE has been used in computational works to predict TSS, along with other methods like ChIPseq [34] and ATACseq [35] to filter out the noise from the signal. The source of noise being the capping sites that do not coincide with promoter regions. There are capping sites in non-promoter regions like RNA splicing sites, which occur when the RNA is recapped [16,36,37]. Some studies attribute approximately 25% of the total capping sites that lie in the downstream of TSS. So, predicting only the TSS site is not enough for mapping of all the capping sites. CAGE sites do not usually coincide to TSS signals like TATA boxes and initiators and the average distance of CAGE peaks to the TATA boxes and initiators were found to be 260 and 91 respectively among the sequences in which these motifs were identified. This requires the context window to be large enough to capture the TSS signal regions and the adjacent regions in order to predict capping. Our method takes a context window of 512 nts and predicts the occurrence of capping with an LOCOCV accuracy of 79.12 ± 1.16%, a precision of 78.64 ± 2.10%, a recall of 77.66 ± 2.11%, along with an F1 score of 78.11 ± 1.11%. On a larger context window of 1024, our method predicts capping with an LOCOCV average accuracy of 79.52 ± 1.17%, an average precision of 79.03 ± 2.04%, an average recall of 77.72 ± 2.32%, and an average F1-score of 78.33 ± 1.21%. Similarly, on fine-tuning on the mouse genome mm9, our method predicts the occurrence of capping with an LOCOCV accuracy of 78.09 ± 1.08%, a precision of 79.78 ± 3.32%, a recall of 73.105 ± 3.36%, and an F1 score of 76.17 ± 1.23%. It also returns regions of interest within the sequence having the maximum attention score. This region of interest has often shown co-occurrence to initiator signals and downstream promoter elements, with positive peaks mapping to initiators 0.133 times and to DPEs 0.578 times per positive case, and negative peaks mapping to initiators 0.231 times and to DPEs 0.590 times per negative case. Our model finds the capping regions in an input genomic sequence along with the tokens corresponding to the highest attention scores, from which we obtain the differentiating motifs for capping. These tokens have been found to be statistically significant when compared to the entire context length and against each other. Some of the prominent differentiating motifs found from the attention scores are TCTTGAAT, GTCTTGAA, ACTCATGT, CACTCATG, CCCTCAGC, TGATCTGC, TTTTTATT, CAATACAT, GATGTGAG, TTGATCTG, AATACATA, ATTTAGGG, TAATTTCA, CTTGAATG, ATTCATAG, and ATGTGAGT, all of which have motif-wise p-values across predicted classes less than 0.01. Out of these motifs, TTTTTATT and ATGTGAGT are also biologically relevant since their resident sites match closely with some TF motifs.

## Supplementary Material

SI_RNAcapping_v2.pdf

## Data Availability

The entire code of this work is available at the following site: https://github.com/DibyoKgpIIT/CAGE_capping/tree/main.
