# Supplementary material for: A transformer-based method for the cap analysis of gene expression and gene expression tag associated capping region prediction in RNA
Source: RNA Biol. 2026 Feb 10;23(1):1–15. doi: 10.1080/15476286.2026.2629530 (PMC12915862; doi:10.1080/15476286.2026.2629530)
Supplement: SI_RNAcapping_v2.pdf [file KRNB_A_2629530_SM4582.pdf]

# Supporting Information

# A Transformer based method for the Cap Analysis of Gene Expression and Gene Expression Tag associated capping region prediction in RNA

Dibya Kanti Haldar[1], Avik Pramanick[2], Chandrama Mukherjee[3], Pralay Mitra[2,*]

[1]Centre for Computational and Data Sciences, Indian Institute of Technology Kharagpur, West Bengal, India-721302

[2]Department of Computer Science and Engineering, Indian Institute of Technology Kharagpur, West Bengal, India-721302

[3]Institute of Health Sciences, Presidency University, Kolkata, West Bengal, India - 700073

*Correspondence to

Pralay Mitra, Ph.D.

Department of Computer Science and Engineering,

Indian Institute of Technology Kharagpur West

Bengal - 721302, India

Email: pralay@cse.iitkgp.ac.in

Phone: +91-3222-282344

ORCID ID: 0000-0003-4119-3788

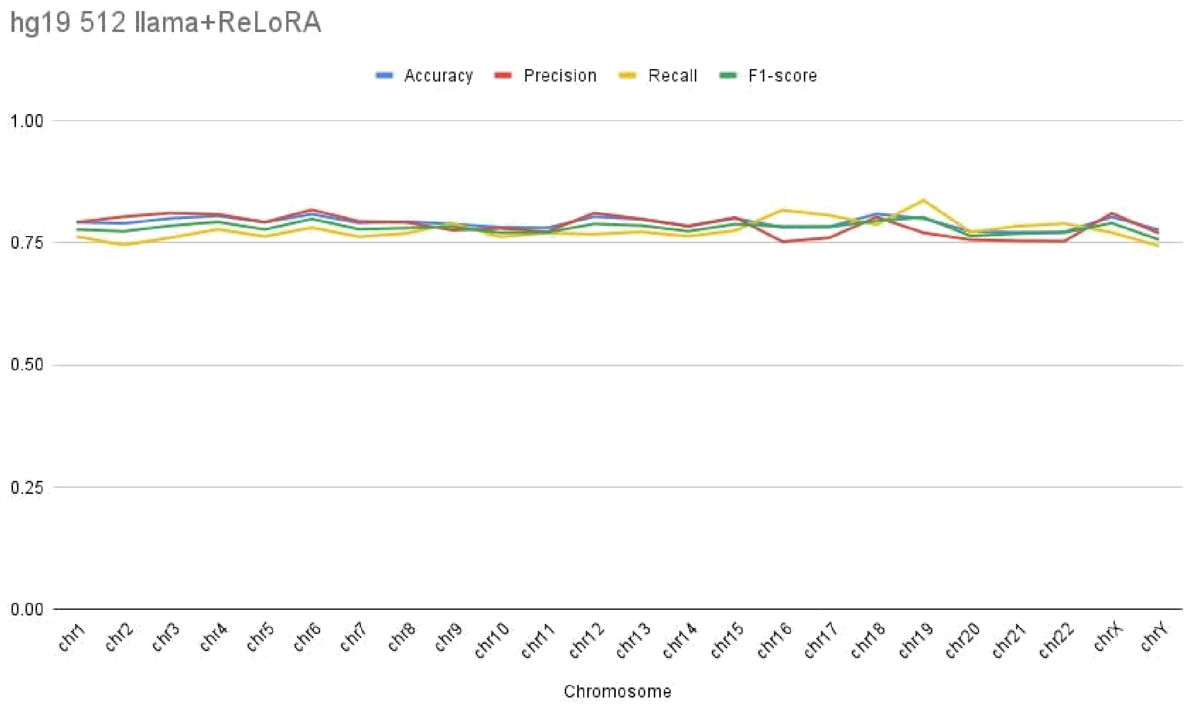


**Figure S1.** Plot showing the performance across metrics like accuracy, precision, recall, and F1-score validated on left out chromosomes 1 through 24 of the human genome hg19 using our Llama and ReLoRA based model.

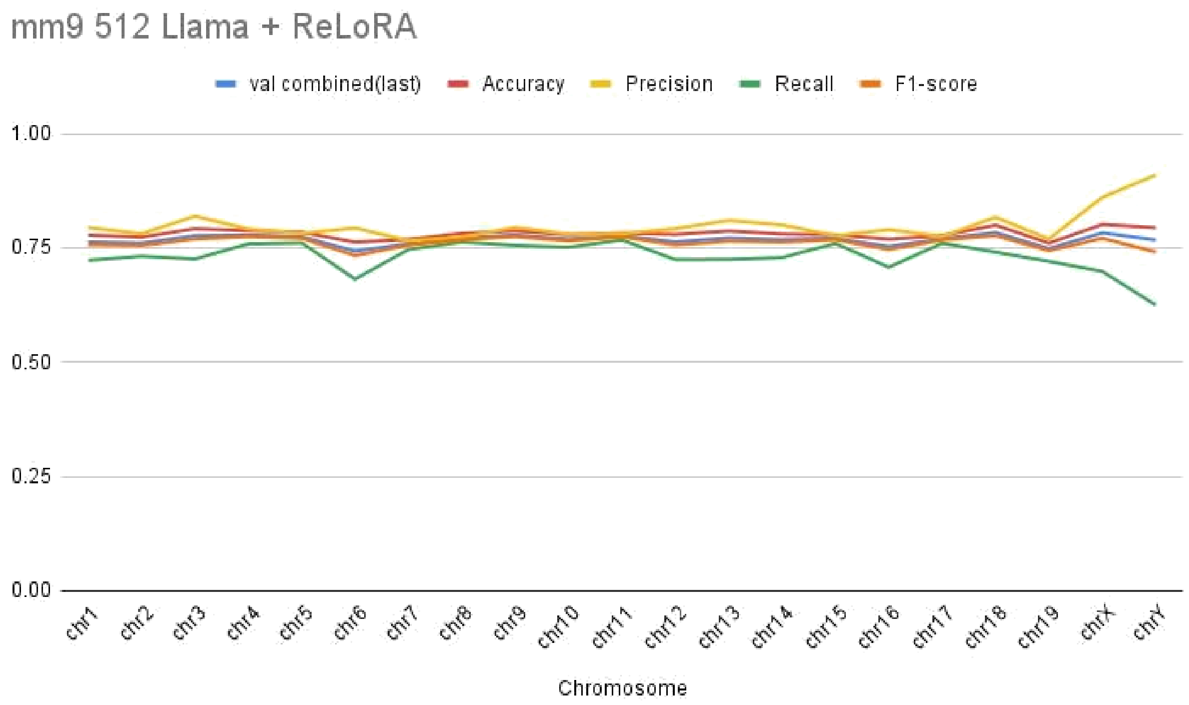


**Figure S2.** Plot showing the performance across metrics like accuracy, precision, recall, and F1-score validated on left out chromosomes 1 through 21 of the mouse genome mm9 using our Llama and ReLoRA based model.

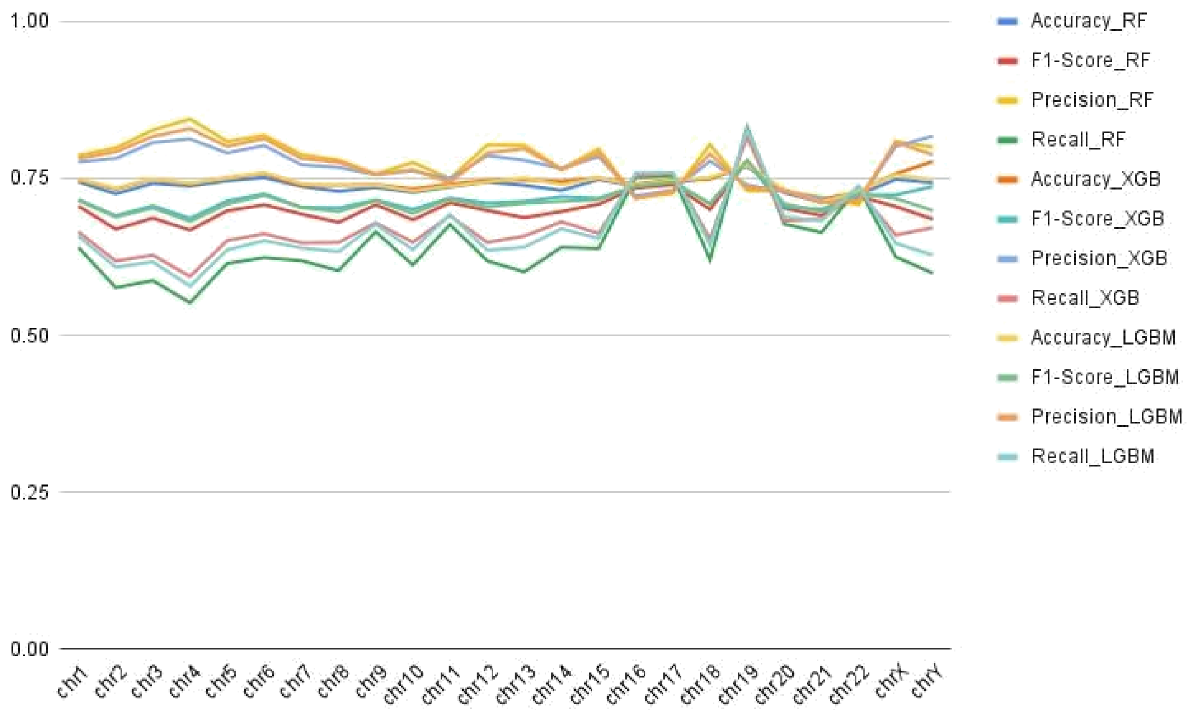


**Figure S3.** Comparison between Random Forest, XG Boost, and Light GBM in different metrics for LOCOCV on human genome hg19 using a context window of size 512.

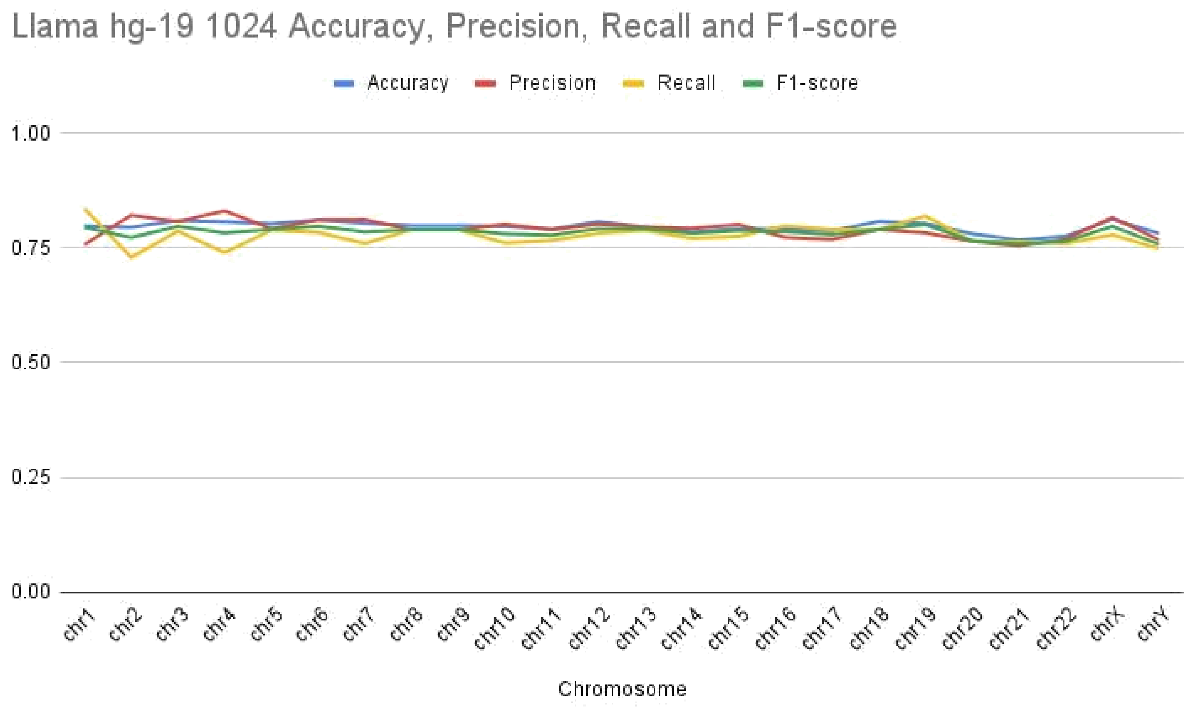


**Figure S4.** Llama 20 million model metrics for LCOCV on human genome hg19 for a context window of size 1024.

## Section ReLoRA

### Pre-training Paradigm

The ReLoRA based pre-training is divided into two steps. A warm start without ReLoRA is followed by a full pre-training with ReLoRA. A small warm start of 1000 steps have been taken during our pre-training.

After every 2000 steps of ReLoRA , 100 steps of warmup without ReLoRA is carried out. Figure S5 demonstrates the ReLoRA pre-training loss curves during the warm start initialization. Here, we can infer a logarithmic reduction in loss values. We follow it up with the ReLoRA pre-training regime, where we can see loss curves with two sudden steep declines, as can be inferred from Figure S6. Figure S7 depicts our learning rate scheduling, and it features a steep climb and a gradual decline, interspersed with periodic V-shaped 100 step cyclic ReLoRA warm-ups which use lower learning rates every 2000 steps.

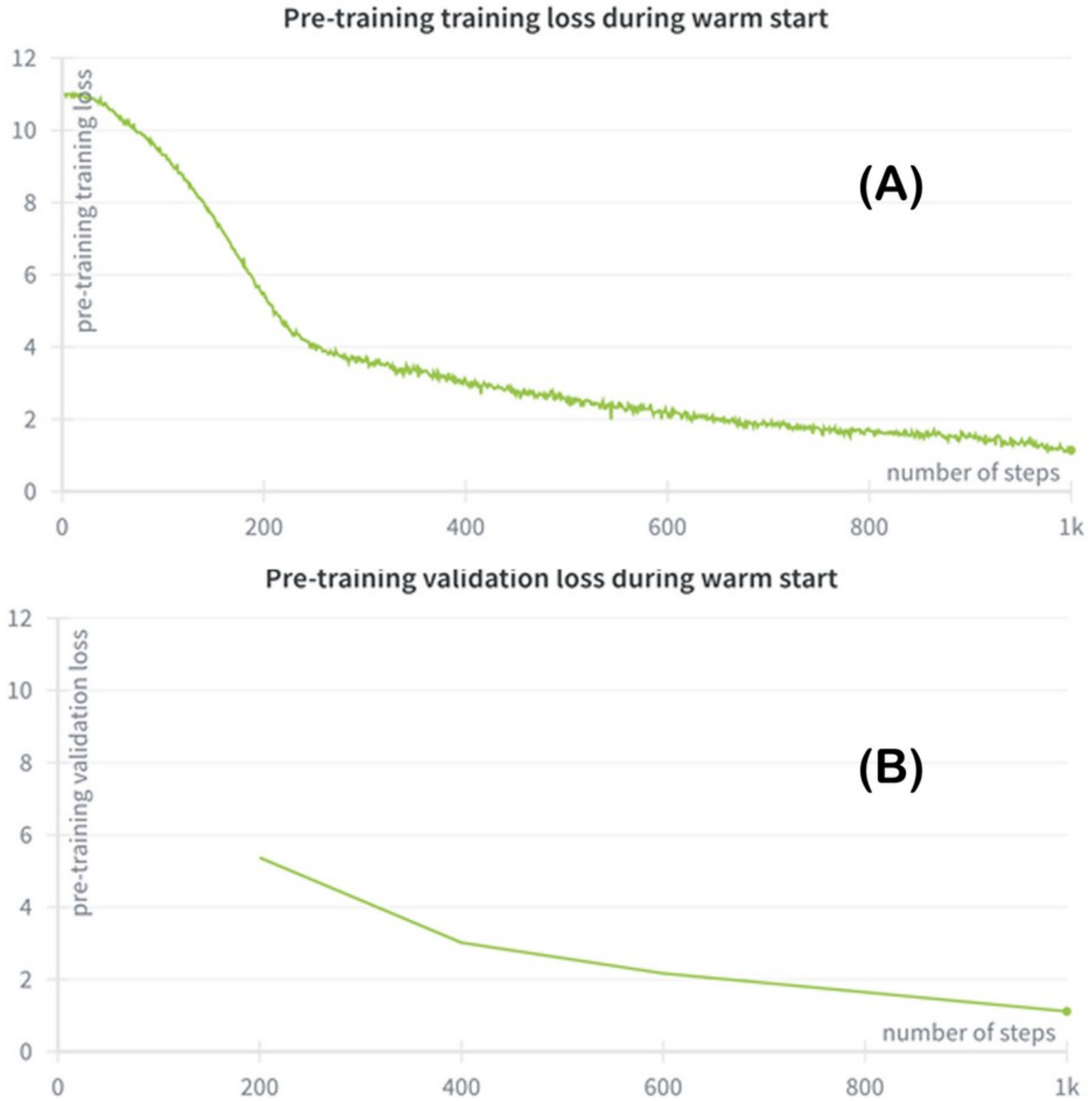


**Figure S5.** The ReLoRA and Llama_for_causal_LM based pre-training during warm start for A. training and B. validation loss curves showing logarithmically reducing losses for both the curves over 1000 steps.

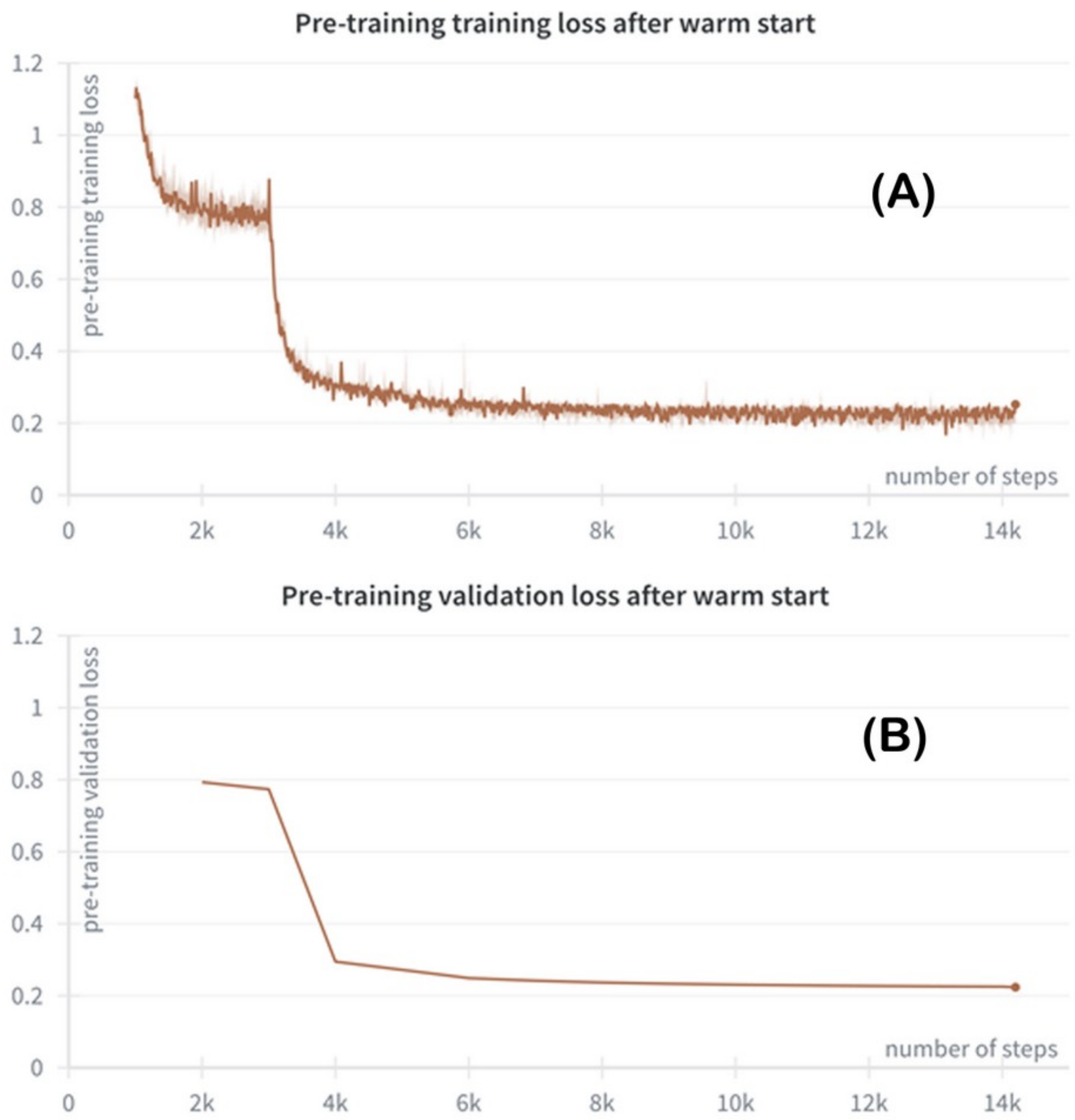


**Figure S6.** The ReLoRA and Llama_for_causal_LM based pre-training following warm start for A. training and B. validation loss curves showing unevenly reducing losses with sudden steep declines for both the curves.

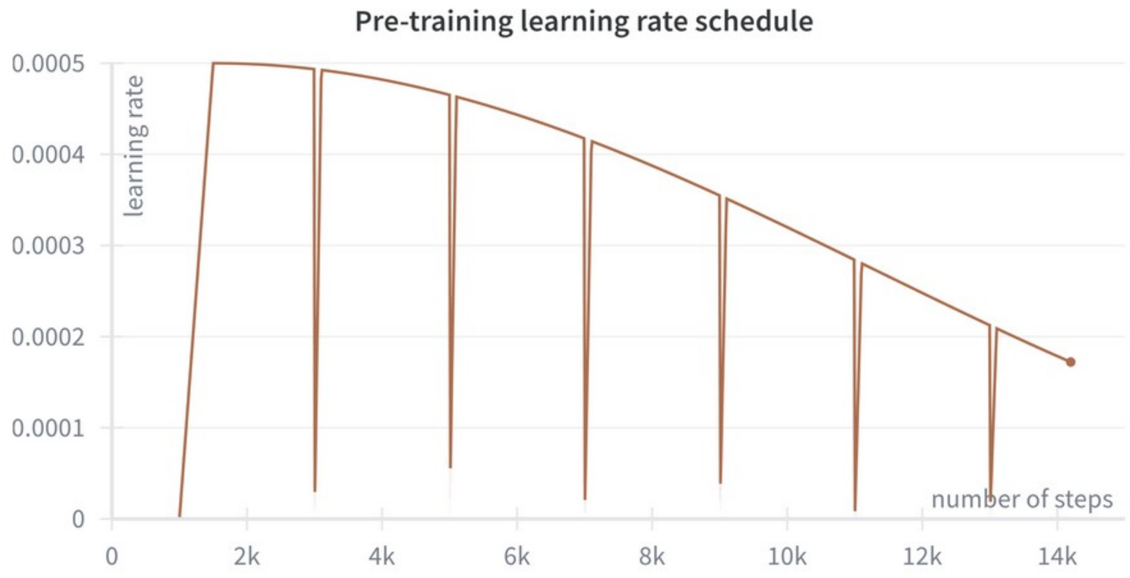


**Figure S7.** The learning rate regime followed during ReLoRA based pre-training following the warm-start. We can see a steep ascent followed by a gradual decline periodically interrupted by steep V-pulses during the periodic ReLoRA warm ups.
